# Reliability and Validity of the Chinese Version of the ERS-ACA Scale Among Chinese College Students

**DOI:** 10.3390/bs16091661

**Published:** 2026-09-16

**Authors:** Xiyue Yu, Xinyu Luo, Sina Zhang, Qingfei Chen, Jialin Fan

**Affiliations:** 1 School of Psychology, Shenzhen University, Shenzhen 518060, China; 2023341091@email.szu.edu.cn (X.Y.); 2022081018@email.szu.edu.cn (X.L.); 2515168006@email.szu.edu.cn (S.Z.); chenqf@szu.edu.cn (Q.C.); 2 School of Artificial Intelligence, Shenzhen University, Shenzhen 518060, China; 3 Shenzhen Humanities and Social Sciences Key Research Bases of the Center for Mental Health, Shenzhen 518000, China; 4 Key Laboratory of Brain Cognition and Emotional Health of Guangdong Higher Education Institutes, Shenzhen University, Shenzhen 518060, China

**Keywords:** ERS-ACA Scale, reliability, validity, college students

## Abstract

Chinese university students face academic, employment, and interpersonal pressures that may increase their vulnerability to anxiety and depression. Artistic creative activities (ACAs) provide contexts in which individuals may regulate emotions, but the strategies used during these activities require appropriate measurement. A previous Chinese adaptation of the Emotion Regulation Strategies for Artistic Creative Activities (ERS-ACA) Scale was validated among art and design majors and omitted one item. The present study evaluated the psychometric properties of the original 18-item Chinese ERS-ACA in 885 university students from multiple academic disciplines. Participants completed the ERS-ACA, the Emotion Regulation Questionnaire (ERQ), and the Student Well-Being Process Questionnaire (Student-WPQ). Confirmatory factor analysis supported the original three-factor structure. Internal consistency was satisfactory for the total score (Cronbach’s alpha = 0.903) and the three subscales (alpha = 0.796–0.814). The three ERS-ACA dimensions were positively associated with cognitive reappraisal, positive well-being, and positive personality, whereas their associations with expressive suppression were small or non-significant. These findings support the structural validity, criterion-related validity, and internal consistency of the 18-item Chinese ERS-ACA in the present university student sample. Further validation in other populations is warranted.

## 1. Introduction

Chinese university students encounter multiple demands during a developmental period marked by increasing academic expectations, competition for postgraduate opportunities, uncertainty in the employment market, and the need to establish new interpersonal networks. These features of social transformation and educational competition can expose students to sustained academic, employment, and social pressures (Cao, 2025; C. Li & Gao, 2024). When such demands exceed perceived coping resources, persistent stress may contribute to anxiety, depressive symptoms, and difficulties in psychological adjustment. The substantial prevalence of depression and poor mental health reported among Chinese university students therefore highlights the importance of understanding processes that may support emotional adjustment in this population (J. Fan et al., 2024; Gao et al., 2020; Wang et al., 2020). Meta-analytic evidence on psychological interventions for depression in Chinese university students further underscores the practical importance of identifying modifiable psychological processes in this population (Fu et al., 2020).

Because many academic and social stressors cannot be immediately removed, how students monitor and regulate their emotional responses may influence their psychological adjustment. Emotion regulation (ER) refers to the processes through which individuals influence which emotions they experience, when they experience them, and how these emotions are expressed (Gross, 1998, 2001). Effective ER can support adaptive functioning, whereas inflexible regulatory patterns are associated with a range of psychological difficulties (Kashdan & Rottenberg, 2010; Thompson & Calkins, 1996). ER strategies may also depend on the activity in which regulation occurs. Activity-specific contexts, including music-making, visual art, expressive writing, dance, and other forms of creative engagement, may afford regulatory processes that are not fully captured by measures of habitual ER in daily life.

Artistic creative activities (ACAs) include activities such as making music, creating visual art, expressive writing, performing, dancing, photography, and crafts. Active arts engagement has been associated with emotional and psychological outcomes across several modalities (Stuckey & Nobel, 2010; Yap et al., 2017). In university-student contexts, arts-based approaches involving music, group painting, and dance have also been examined in relation to anxiety, depressive symptoms, self-concept, and self-esteem (X. Fan & Zhang, 2018; Tian & Tian, 2019; Yan et al., 2018). Clinical arts-therapy research likewise provides evidence that creative engagement can be related to emotional expression and psychological well-being (Haeyen et al., 2018; Puig et al., 2006). These findings should not be interpreted as showing that every ACA or arts-based intervention produces the same outcome. More importantly for the present study, evidence of emotional benefits does not by itself identify the regulatory strategies used during creative engagement. Van Goethem and Sloboda’s (2011) framework distinguishes the goal of regulation, the selected activity or tactic, the strategy used during the activity, and the mechanism through which emotional change occurs. This distinction highlights the need for instruments that specifically assess ER strategy use within artistic creative contexts.

Emotion regulation measures differ in both contextual breadth and art-form specificity. Context-general instruments such as the Emotion Regulation Questionnaire (ERQ) assess habitual cognitive reappraisal and expressive suppression across everyday situations (Gross & John, 2003), but they do not indicate which categories of strategies individuals use specifically during ACA. Content-specific evidence from music illustrates why this distinction matters. Listening to music can itself influence cognitive and affective states; for example, both traditional Chinese music and Western classical music attenuated fatigue-related declines in alertness and hedonic tone after a cognitive task (Tan et al., 2026). However, effects of listening to a particular type of music do not identify the strategies individuals use when they actively create art. Artistic creative activities are multimodal and can involve shared processes such as imagination, cognitive and emotional stimulation, skill cultivation, and regulatory processes related to distraction, reappraisal, problem-solving, self-identity, self-esteem, and agency (Fancourt et al., 2019; Fancourt & Ali, 2019). These processes may extend beyond those captured by exposure-focused or single-modality measures. Correspondingly, art-form-specific instruments such as the Music in Mood Regulation Scale assess regulation within a single artistic modality and are not designed to assess strategy use across visual art, expressive writing, performing arts, and other forms of ACA (Saarikallio, 2008). This creates a measurement gap: an arts-based ER measure should be specific to active artistic creation while remaining applicable across different art forms. Such a measure would complement, rather than replace, context-general ER instruments and content-specific measures of individual art forms.

The Emotion Regulation Strategies for Artistic Creative Activities (ERS-ACA) Scale was developed to assess the categories of ER strategies individuals use while engaging in ACAs (Fancourt et al., 2019). Grounded in theoretical work that conceptualizes ER as involving diverse processes operating across emotional situations (Gross, 1998, 2001), the ERS-ACA comprises an overall score and three dimensions that represent categories of related processes rather than three isolated strategies. Avoidance strategies include distraction, suppression, and detachment, as illustrated by the item ‘I can block out any unwanted thoughts or feelings.’ Approach strategies include acceptance, reappraisal, and problem-solving, as illustrated by ‘It helps me to put worries or problems I have in perspective.’ Self-development strategies involve self-identity, self-esteem, and agency, as illustrated by ‘It makes me feel stronger in myself.’ The 18-item scale therefore provides a cross-art-form measure of ER strategy categories used during creative engagement.

The original validation supported an 18-item structure consisting of seven avoidance items, six approach items, and five self-development items, with strong internal consistency and evidence of convergent, divergent, and test–retest reliability (Fancourt et al., 2019). Yu and Shi (2025) subsequently developed a Chinese adaptation for art and design students. Their item analysis identified a low item–total correlation for Item 1 (r = 0.233), and the resulting 17-item version showed satisfactory factor structure and reliability. The present study complements that work by examining whether the original 18-item structure is supported in a sample drawn from multiple academic disciplines. The earlier Chinese adaptation used the Self-expression and Emotion Regulation in Art Therapy Scale (SERATS) as an external criterion; SERATS was originally developed in a clinical art-therapy context for people with personality disorders (Haeyen et al., 2017). The external measures in the present study were therefore selected to provide complementary evidence and to position ACA-related strategy categories within a broader network of ER and student psychological functioning. The original ERS-ACA validation used the ERQ to examine whether ACA-related strategies were distinct from habitual ER in daily life (Fancourt et al., 2019). Cognitive reappraisal is conceptually represented within approach-oriented processes, whereas expressive suppression overlaps only partly with the broader avoidance category, which also contains distraction and detachment. The Student Well-Being Process Questionnaire (Student-WPQ) assesses student well-being and positive psychological functioning (Williams et al., 2017). Positive well-being and positive personality were therefore used to examine whether ERS-ACA scores were associated with theoretically relevant aspects of student functioning.

Accordingly, the primary aim of this study was to evaluate the psychometric properties of the original 18-item Chinese ERS-ACA among a broad sample of Chinese university students. Specifically, we examined whether the original three-factor structure was supported, assessed the internal consistency of the total scale and its subscales, and evaluated criterion-related validity through associations with habitual cognitive reappraisal and expressive suppression as well as positive well-being and positive personality. In this way, the study examined whether the complete 18-item scale could be retained in a university sample extending beyond students in art and design majors.

## 2. Materials and Methods

### 2.1. Procedures

#### 2.1.1. Data Collection

Participants were recruited from Chinese university-student populations through Chinese social media platforms (e.g., WeChat and Rednote) over a one-month period using a non-probability, voluntary-response sampling approach. This study was approved by the Research Ethics Committee of Shenzhen University (SZU_PSY_2025_065). All data were analysed anonymously, and all participants provided informed consent. Participants received a small monetary reward upon completion.

#### 2.1.2. Data Exclusion

To ensure data quality and analytical validity, the raw data (n = 1186) were screened using the following criteria. First, incomplete responses were excluded. Second, responses failing either of the two attention-check items—(1) “For this question, please choose ‘6’” and (2) “For this question, please select ‘Completely Disagree’”—were removed. Third, duplicate responses from the same IP address were removed. Fourth, responses from participants outside the target university-student age range were excluded; the retained sample ranged from 16 to 30 years. Fifth, responses with abnormal completion times (less than 172 s or more than three standard deviations above the mean) were excluded to minimize careless responding.

After applying these criteria, a total of 885 valid responses were retained for analysis (valid response rate: 74.6%).

### 2.2. Participants

A priori power analyses were conducted during study planning to determine the required sample size. For the prespecified three-factor CFA model (df = 132), an RMSEA-based analysis following MacCallum et al. (1996), with RMSEA_0_ = 0.05, RMSEA_1_ = 0.08, and α = 0.05, indicated minimum sample sizes of 110 and 137 for 0.80 and 0.90 power, respectively. A complementary G*Power 3.1 analysis (Faul et al., 2009) for the planned pairwise criterion-validity correlations, assuming a two-tailed test, α = 0.05, and r = 0.20, indicated required sample sizes of 193 and 258 for 0.80 and 0.90 power, respectively. The final analytic sample of 885 exceeded these requirements.

The demographic information of valid participants was summarized across six aspects: gender, age, academic level, major, health status, and place of residence. The sample comprised 260 males (29.4%) and 625 females (70.6%), aged 16 to 30 years (M = 20.60, SD = 1.95). Participants represented diverse academic levels and a wide range of disciplines (UNESCO Institute for Statistics, 2012). Health status was recorded as a self-reported demographic item with three response categories (healthy, sub-healthy, and unhealthy); these categories describe participants’ self-classification rather than a clinical diagnosis. Detailed distributions are presented in Table 1. Participants resided in over 150 cities across China, with the largest proportions from Shenzhen (23.8%), Guangzhou (17.4%), and Hangzhou (13.7%); other individual regions each accounted for less than 3% of the sample.

### 2.3. Measures

#### 2.3.1. ERS-ACA Scale

The Emotion Regulation Strategies for Artistic Creative Activities (ERS-ACA) Scale is an 18-item instrument designed to assess the categories of ER strategies individuals use during ACAs. It yields a total score and three subscale scores: avoidance strategies (7 items), approach strategies (6 items), and self-development strategies (5 items). These dimensions represent clusters of related regulatory processes rather than three individual strategies. All items are positively keyed and rated on a 5-point Likert scale; higher mean scores indicate more frequent use of the corresponding category of strategies.

The original validation reported high internal consistency (Cronbach’s α = 0.93 for the total score, 0.90 for avoidance, 0.88 for approach, and 0.88 for self-development) and strong two-week test–retest reliability (r = 0.85, *p* < 0.001), together with convergent and divergent validity evidence (Fancourt et al., 2019). Criterion-related evidence included associations between avoidance and the B-MMRS transference subscale, between approach and stress-release/mental-work components, and moderate associations between self-development and B-MMRS subscales (Fancourt et al., 2019; Saarikallio, 2012).

With the permission of Fancourt et al., the developers of the original English version of the ERS-ACA Scale, we conducted the translation and adaptation of the Chinese version. The process strictly followed the back-translation procedure proposed by Brislin (1970). First, two bilingual researchers with expertise in English independently translated the original English version into Chinese. This Chinese draft was then back-translated into English by two other psychology researchers unfamiliar with the original scale. The team then compared the back-translated version with the original, discussing and revising each item with semantic or cultural issues until consensus was achieved. This process finalized a Chinese version that was both semantically equivalent and culturally appropriate. The pre-final version was piloted on a sample of 17 university students to confirm item comprehensibility, and minor adjustments were made based on feedback before the instrument was finalized.

#### 2.3.2. ERQ

The Emotion Regulation Questionnaire (ERQ), developed by Gross and John (2003), assesses two habitual ER strategies: cognitive reappraisal (6 items) and expressive suppression (4 items). Items are rated on a 7-point Likert scale (1 = strongly disagree to 7 = strongly agree), with higher mean scores indicating more frequent use of the corresponding strategy. In the present sample, Cronbach’s α was 0.790 for cognitive reappraisal and 0.843 for expressive suppression. The ERQ was used as an external criterion for criterion-related validity.

#### 2.3.3. Student-WPQ Scale

The Student Well-Being Process Questionnaire (Student-WPQ) was developed by Williams et al. (2017) to assess student well-being, demand–resource characteristics, personality traits, and coping strategies. It includes a Well-being Subscale (14 items), a Demand–Resource Subscale (13 items), a Personality Traits Subscale (8 items), and a Coping Strategies Subscale (5 items), with items rated on a 10-point scale. In the present study, “positive well-being” refers specifically to the composite score operationalized within the Student-WPQ framework rather than to a single independent dimension. The first three subscales were administered; Cronbach’s α was 0.939 for well-being, 0.801 for demand–resource characteristics, and 0.829 for personality traits. Positive well-being and positive personality were used as external indicators in the criterion-related validity analyses. A related Chinese-language well-being measure derived from the WPQ framework has also been translated using forward- and back-translation procedures and applied in Chinese university-student samples (W. Li et al., 2022), providing supporting evidence for the contextual relevance of WPQ-derived assessment in this population.

#### 2.3.4. Statistical Methods

After data cleaning, descriptive statistics, item analyses, Spearman rank-order correlations, and internal-consistency analyses were conducted in SPSS 27.0.1, and confirmatory factor analysis (CFA) was conducted in R 4.6.1. The hypothesized CFA specified three correlated latent factors: Avoidance Strategies (Q1, Q3, Q4, Q8, Q11, Q14, Q18), Approach Strategies (Q2, Q9, Q10, Q12, Q13, Q16), and Self-development Strategies (Q5, Q6, Q7, Q15, Q17). Each item was specified to load on its intended factor only, with the three latent factors allowed to correlate. Because the Henze–Zirkler test indicated a significant departure from multivariate normality, robust maximum likelihood estimation (MLR) was used. Model fit was evaluated using χ^2^, CFI, TLI, and RMSEA with its 90% confidence interval, and the indices were interpreted jointly rather than as rigid decision rules (Bentler & Bonett, 1980; Browne & Cudeck, 1992; Tucker & Lewis, 1973).

## 3. Results

### 3.1. Item Analysis

Item–total and item–factor correlations were computed to assess item quality. All items were significantly correlated with the total scale score (r = 0.517–0.716, all *p* < 0.001). Each item showed a stronger correlation with its intended subscale (r = 0.626–0.799, all *p* < 0.001) than with the total scale score. Accordingly, all 18 items met the retention criteria and none was removed. The item-level descriptive statistics and item–total correlations are presented in Table 2.

### 3.2. Validity Analysis

#### 3.2.1. Confirmatory Factor Analysis

**Figure 1:**
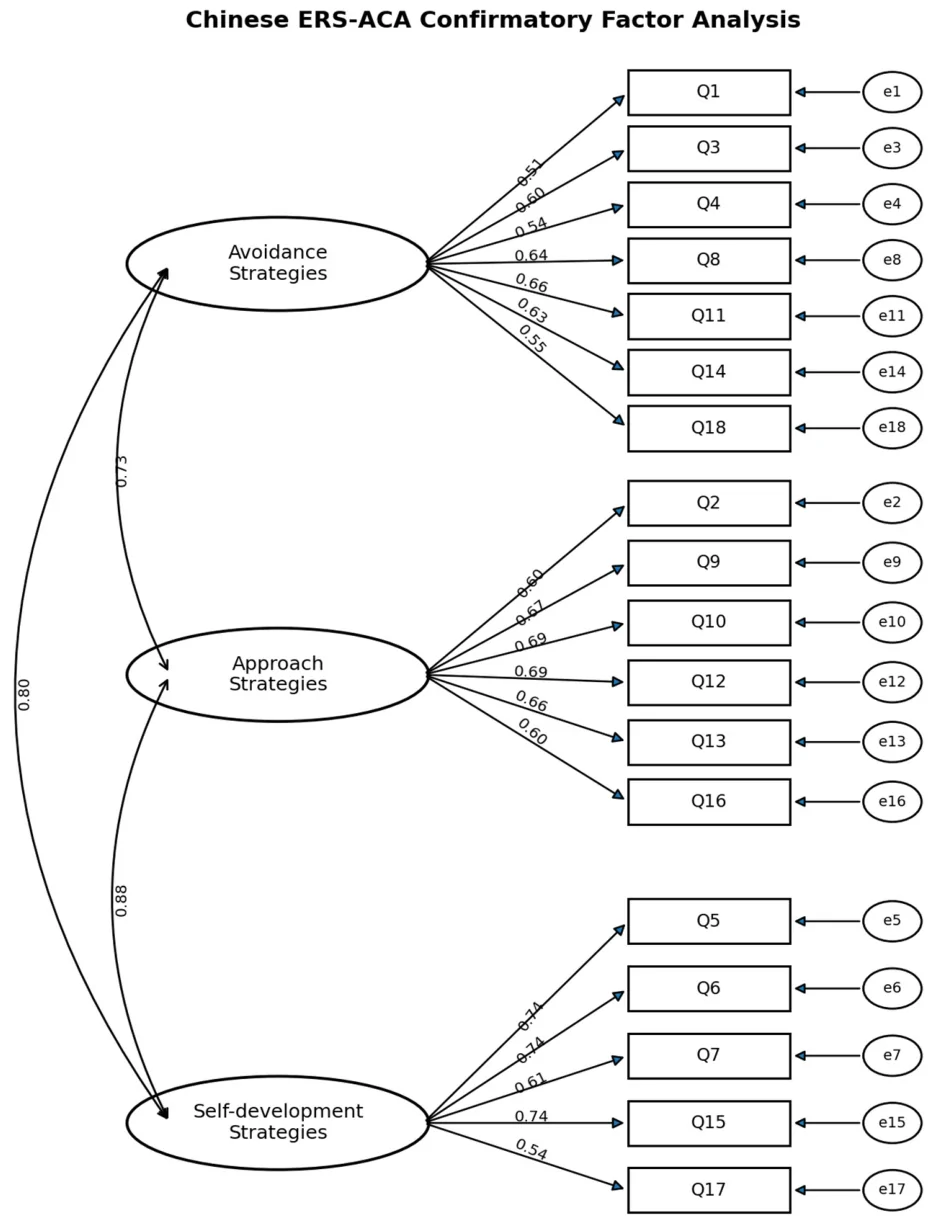
Confirmatory factor analysis results for the Chinese version of the ERS-ACA Scale. Note: Standardized factor loadings are shown on the single-headed arrows; curved double-headed arrows show correlations among the three latent factors. Residual labels correspond to the ERS-ACA item numbers. CFA = confirmatory factor analysis.

Using MLR, the three-factor CFA yielded χ^2^(132) = 521.809, *p* < 0.001, CFI = 0.915, TLI = 0.901, RMSEA = 0.065, 90% CI [.059, 0.070], and SRMR = 0.047. The χ^2^/df ratio was 3.95. The model fit indices are presented in Table 3. The statistically significant χ^2^ was considered alongside the other fit indices because χ^2^ is sensitive to large sample sizes. The standardized three-factor CFA model is presented in Figure 1. Standardized factor loadings were statistically significant (all *p*s < 0.001) and ranged from 0.51 to 0.74: 0.51–0.66 for Avoidance Strategies, 0.60–0.69 for Approach Strategies, and 0.54–0.74 for Self-development Strategies. Q1 had the lowest loading (0.51) but remained positively associated with its intended factor. The latent factors were substantially correlated: avoidance with approach, r = 0.73; avoidance with self-development, r = 0.80; and approach with self-development, r = 0.88. Taken together, the fit indices, item loadings, and factor correlations supported the hypothesized 18-item, three-factor structure in the present sample.

#### 3.2.2. Criterion Validity

Criterion-related validity was examined using the ERQ and selected Student-WPQ indicators as external criteria. Given the non-normal distributions, Spearman’s rank-order correlation was used as a non-parametric alternative to Pearson correlation because it does not require normally distributed variables and is appropriate for monotonic associations. Correlations were examined with ERQ cognitive reappraisal and expressive suppression and with Student-WPQ positive well-being and positive personality. The full pattern of coefficients is reported in Table 4.

As shown in Table 4, all three ERS-ACA subscales were positively associated with cognitive reappraisal: approach (Spearman’s *ρ* = 0.536, *p* < 0.001), self-development (*ρ* = 0.463, *p* < 0.001), and avoidance (*ρ* = 0.458, *p* < 0.001). Expressive suppression was not significantly associated with avoidance (*ρ* = −0.048) and showed only small negative associations with approach (*ρ* = −0.084, *p* < 0.05) and self-development (*ρ* = −0.114, *p* < 0.001).

For the Student-WPQ indicators, all three ERS-ACA subscales were positively correlated with positive well-being and positive personality. Self-development showed the largest correlations with positive well-being (Spearman’s *ρ* = 0.568, *p* < 0.001) and positive personality (*ρ* = 0.526, *p* < 0.001), whereas avoidance showed the smallest corresponding coefficients (*ρ* = 0.479 and 0.425, respectively; both *p* < 0.001).

### 3.3. Reliability Analysis

The Chinese ERS-ACA showed satisfactory internal consistency. Cronbach’s α was 0.903 for the total score and ranged from 0.796 to 0.814 across the three subscales (Table 5).

## 4. Discussion

The present study evaluated the psychometric properties of the original 18-item Chinese ERS-ACA in a broad sample of university students. The discussion therefore focuses on the scale’s factor structure, internal consistency, criterion-related associations, and its relationship to previous validation work. Overall, the findings supported the original three-factor structure and provided evidence that the full 18-item scale can be used to assess avoidance, approach, and self-development strategy categories in the present sample.

### 4.1. Reliability and Validity Characteristics of the ERS-ACA Scale

Confirmatory factor analysis supported the three-factor structure comprising avoidance, approach, and self-development strategies, with acceptable fit indices (CFI = 0.915, TLI = 0.901, and RMSEA = 0.065). Internal consistency was satisfactory for the total score and the three subscales. The positive associations with cognitive reappraisal, positive well-being, and positive personality further supported criterion-related validity. These results provide evidence for the structural validity and reliability of the Chinese ERS-ACA in the current sample of university students; they do not, by themselves, establish cultural equivalence or measurement invariance across populations.

### 4.2. Research Findings

The associations with the ERQ were consistent with the conceptual distinction between habitual ER in daily life and ER strategy categories used during ACAs. Approach showed the strongest positive association with cognitive reappraisal, which is expected because reappraisal is one of the processes represented within the approach category. Avoidance and self-development also showed positive associations with reappraisal, suggesting that habitual reappraisal may relate to several forms of regulatory engagement during artistic creation without being equivalent to any single ERS-ACA dimension.

Expressive suppression showed no significant association with avoidance and only small negative associations with approach and self-development. This limited overlap is theoretically plausible because the ERQ expressive-suppression subscale assesses a habitual tendency to inhibit emotional expression, whereas the ERS-ACA avoidance category also includes distraction and detachment. This distinction is consistent with evidence that disengagement-oriented strategies are heterogeneous and that positive distraction can function differently from avoidance (Waugh et al., 2020). The findings therefore suggest that everyday expressive suppression and ACA-related avoidance should not be treated as interchangeable constructs.

The positive associations with positive well-being and positive personality place the ERS-ACA dimensions within a broader network of student psychological functioning. Self-development showed the largest correlations with both indicators, consistent with its emphasis on self-identity, self-esteem, and agency. These correlational findings support criterion-related validity but do not demonstrate that ACA strategy use causes improvements in well-being or personality.

### 4.3. Research Contribution

The present findings complement the earlier Chinese adaptation by Yu and Shi (2025). That study focused on art and design students and omitted Item 1 after it showed a low item–total correlation. In the present, broader university sample, Item 1 showed acceptable associations with both the total score and its intended subscale, and the complete 18-item model received support. Differences between the two studies may reflect sample composition and analytic decisions. Taken together, the studies provide evidence for related applications of the ERS-ACA in Chinese student populations rather than implying that one adaptation invalidates the other.

The inclusion of students from multiple academic disciplines extends evidence beyond samples restricted to art and design majors. In addition, using the ERQ and Student-WPQ enabled the present study to examine relationships with habitual ER and student psychological functioning. These measures complement the art-specific criterion used in the earlier adaptation and provide a different perspective on the scale’s criterion-related validity.

### 4.4. Limitations and Future Directions

Despite the positive findings, several limitations should be considered. First, the sample consisted exclusively of university students aged 16–30 years and showed a marked gender imbalance (625 females and 260 males). This relatively homogeneous educational context and demographic composition may limit generalizability. The present findings therefore support the scale only in this sample and should not be interpreted as evidence of applicability to working adults, clinical populations, or other cultural groups. Future studies should recruit broader, more representative samples and test measurement invariance across relevant populations.

Second, the study relied on self-report measures, which may introduce common-method variance and may not capture implicit regulatory processes. Future studies could incorporate behavioural observations, interviews, or experimental paradigms to provide complementary validation evidence.

Third, the absence of a test–retest reliability assessment limits knowledge about the temporal stability of the ERS-ACA. Future studies should include a 2- to 4-week retest design to evaluate the scale’s consistency over time. 

Fourth, cultural values and self-construal were not directly measured. Consequently, the present findings should not be interpreted as evidence of cultural mechanisms or cross-cultural measurement equivalence. Future research could assess relevant cultural variables and conduct measurement-invariance analyses across populations.

Fifth, the study did not document participants’ frequency or type of artistic creative activity engagement and did not test score differences across academic majors. Consequently, the present data cannot determine whether ERS-ACA scores or psychometric properties vary by ACA engagement pattern or field of study. Future studies should record the forms and frequency of ACA participation and examine relevant subgroup differences.

In addition, future studies could explore the specificity of the ERS-ACA by measuring its correlation with established scales, such as the Brief Music in Mood Regulation Scale (B-MMRS) (Saarikallio, 2012) and the Difficulty in Emotion Regulation Scale (DERS) (Gratz & Roemer, 2004). Longitudinal or intervention studies could also be conducted to investigate the long-term effects and causal mechanisms through which art-based ERSs influence mental health. Such research would further demonstrate the practical value of the ERS-ACA in promoting emotional well-being among college students.

## 5. Conclusions

Overall, this study provides evidence for the structural validity, criterion-related validity, and internal consistency of the original 18-item Chinese ERS-ACA in the present sample of university students. The findings support the three-factor structure of avoidance, approach, and self-development strategies and show that ACA-related strategy categories are associated with, but distinct from, habitual ER and positive student functioning. The results also complement the previous 17-item Chinese adaptation by demonstrating that the complete 18-item structure was supported in a sample spanning multiple academic disciplines. Further studies are needed to examine temporal stability, measurement invariance, and generalizability beyond university populations.

## Figures and Tables

**Table 1:** Demographic Characteristics of the Valid Participant Group.

		Effective Sample (n = 885)	
		n	%
Gender	Male	260	29.4
	Female	625	70.6
Academic level	Doctoral Degree	30	3.4
	Master’s Degree	76	8.6
	Bachelor’s Degree	722	81.6
	Associate Degree	57	6.4
Major	Education	92	10.4
	Humanities and arts	146	16.5
	Social Sciences, Business and Law	181	20.5
	Science	124	14.0
	Engineering, Manufacturing and Construction	201	22.7
	Agriculture	18	2.0
	Health and Welfare	85	9.6
	Other	38	4.3
Health status	Healthy	484	54.7
	Sub-healthy	386	43.6
	Unhealthy	15	1.7

**Table 2:** Item Analysis of the ERS-ACA Scale.

Factor	Item	M ± SD	r
Avoidance Strategies	Q1: I can block out any unwanted thoughts or feelings	3.35 ± 0.94	0.555 **
	Q3: I can shake off any anxieties in my life	3.68 ± 0.90	0.596 **
	Q4: I feel I am in my own little bubble, away from ordinary worries	2.89 ± 1.08	0.517 **
	Q8: It helps me forget about my worries	3.53 ± 1.02	0.608 **
	Q11: It helps me to disengage from things that are bothering me	3.68 ± 0.93	0.672 **
	Q14: It makes me feel detached from negative things in my life	3.74 ± 0.85	0.601 **
	Q18: It redirects my attention so I forget unwanted thoughts and feelings	3.92 ± 0.85	0.518 **
Approach Strategies	Q2: I can contemplate what is going on in my life with a clear mind	3.84 ± 0.84	0.610 **
	Q9: It helps me refocus on what matters in my life	3.84 ± 0.83	0.641 **
	Q10: It helps me to come to terms with my own emotions	3.91 ± 0.85	0.669 **
	Q12: It helps me to put worries or problems I have in perspective	3.47 ± 0.97	0.654 **
	Q13: It helps me to understand my own feelings about things that are on my mind	3.64 ± 0.89	0.616 **
	Q16: It makes me reflect on my emotions	3.85 ± 0.82	0.569 **
Self-development Strategies	Q5: I feel more confident in myself	3.63 ± 0.93	0.716 **
	Q6: It boosts my self-esteem	3.69 ± 0.91	0.694 **
	Q7: It gives me a sense of purpose	3.71 ± 0.92	0.599 **
	Q15: It makes me feel stronger in myself	3.42 ± 0.94	0.693 **
	Q17: It reaffirms my identity	3.98 ± 0.84	0.575 **

Note: Q1 to Q18 represent the 18 ERS-ACA items; M = mean; SD = standard deviation; r = Pearson item–total correlation coefficient. ** *p* < 0.001.

**Table 3:** Model Fit Indices of the ERS-ACA Scale.

	Chi-Square	df	CFI	TLI	RMSEA	SRMR	χ^2^/df
Acceptable Threshold	-	-	≥0.90	≥0.90	≤0.08	<0.08	<5
ERS-ACA	521.809	132	0.915	0.901	0.065 [0.059, 0.070]	0.047	3.95

**Table 4:** Correlations Between the ERS-ACA Scale and Factors of the ERQ and Student-WPQ Scales.

	Avoidance Strategies	Approach Strategies	Self-Development Strategies
Cognitive reappraisal	0.458 **	0.536 **	0.463 **
Expressive suppression	−0.048	−0.084 *	−0.114 **
Positive well-being	0.479 **	0.547 **	0.568 **
Positive personality	0.425 **	0.478 **	0.526 **

Note: Rows represent ERQ and Student-WPQ criterion variables; columns represent ERS-ACA subscales. Values are Spearman’s *ρ*. ** *p* < 0.001; * *p* < 0.05.

**Table 5:** Reliability Analysis of the Chinese Version of the ERS-ACA Scale.

Factor	Cronbach’s α	Standardized Cronbach’s α	Number of Items
Avoidance Strategies	0.796	0.798	7
Approach Strategies	0.814	0.815	6
Self-development Strategies	0.798	0.800	5
Total	0.903	0.904	18

## Data Availability

The data supporting the findings of this study are currently embargoed in a private repository on the Open Science Framework (OSF). The data will be made publicly available with a permanent DOI upon acceptance of the manuscript for publication. Data are however available from the authors upon reasonable request and with permission.

## Abbreviations

The following abbreviations are used in this manuscript:

ERS-ACA	Emotion Regulation Strategies for Artistic Creative Activities
ER	Emotion Regulation
ERQ	Emotion Regulation Questionnaire
ACAs	Artistic Creative Activities
